# Behavioral and Neural Changes Induced by a Blended Essential Oil on Human Selective Attention

**DOI:** 10.1155/2019/5842132

**Published:** 2019-10-15

**Authors:** Jieqiong Liu, Shi Cai, Danni Chen, Ke Wu, Yang Liu, Ruqian Zhang, Mei Chen, Xianchun Li

**Affiliations:** ^1^School of Psychology and Cognitive Science, Shanghai Changning-ECNU Mental Health Center, East China Normal University, Shanghai 200062, China; ^2^Daikin (China) Investment Co., Ltd., Shanghai 200062, China

## Abstract

Selective attention refers to the selecting and preferential processing of specific information while simultaneously suppressing irrelevant distractors, activities linked to various cognitive skills and academic achievements. The influence of essential oils on the cognition of humans has been extensively explored. However, the effects of essential oils on human selective attention and the underlying neural mechanisms remain unclear. In the present study, participants were divided into a “blended essential oil” group and a “no essential oil” group and enrolled on a negative priming task, including a control condition and a negative priming condition. The event-related potential technique was used to examine the brain mechanisms underlying the blended essential oil effects on human selective attention. Behavioral results showed that individuals responded more quickly in the negative priming condition when exposed to the blended essential oil. In addition, the blended essential oil eliminated the differences in the P300 amplitude in the postcentral area of the brain between the negative priming condition and the control condition. Moreover, the blended essential oil led to stronger functional connectivity during the task. The present study thus suggests that blended essential oil can significantly change brain activity and functional connections in human beings, which may improve human selective attention.

## 1. Introduction

In daily life, we are presented with large amounts of information, but the capacity of the cognitive system is limited [1]. Therefore, it is essential for us to attend selectively to goal-relevant information while inhibiting distracting or irrelevant information. The mental process through which this is done is called selective attention. Many studies have demonstrated that performance on selective attention tasks is closely correlated with academic skills in general [2–4] and with specific cognitive abilities, including working memory [5], speech processing [6], and nonverbal intelligence [7]. Moreover, selective attention deficiency may be partially involved in certain neural disorders, such as autism spectrum disorder [8] and attention deficit disorder [9]. Therefore, it is important to take suitable measures to improve human selective attention.

Recently, essential oils have become highly popular in the field of alternative medicine. Since ancient times, both physical and mental disorders have been treated with essential oils, including rosemary [10], sage [11], and lavender [12]. Furthermore, many studies have suggested that the inhalation of essential oils improves mood, increases memory, and decreases stress [13–15]. It has been found that some essential oils, containing 1,8-cineole, menthol [16], *β*-pinene [17], and limonene [18], can have a stimulating effect on brain wave activity. One electroencephalography (EEG) study investigated the effect of the San-Jo-In essential oil on human neural activity. The results showed that during inhalation of the San-Jo-In essential oil, the EEG spectrum values of the fast alpha waves were enhanced by 50%, suggesting that the essential oil was able to alter EEG activity and has positive effects on mood and the cognitive functions of the brain [19].

A handful of studies have demonstrated the effects of essential oils on human attention. For example, Shimizu et al. [20] tested the effects of essential oils on individuals' vigilance (referred to here as sustained attention). They found that the increase in reaction time was significantly lower with lavender than with the control, suggesting that lavender essential oil helps to restrain a decrease in vigilance or, in other words, to maintain sustained attention in long-term-vigilance tasks. It also confirmed the sedative effect of lavender on excessive arousal [20]. In another study, 20 older volunteers were randomly exposed to sage essential oil or a placebo, after which their memory and attention performance were measured using the Cognitive Drug Research computerized assessment system. Compared with the placebo condition, 333 mg dose of sage essential oil significantly enhanced accuracy of attention and secondary memory performance [21]. In the current study, we selected a blended essential oil that mainly contained 1,8-cineole, 3-carene, *β*-pinene, *β*-caryophyllene, and carnosic acid. The first four components can enhance the activation of the central nervous system (CNS) [14, 17, 22], while carnosic acid can protect brain cells from oxidative damage and thereby improve memory and cognition [23]. Considering the positive effect of the above-mentioned components on both human attention and brain activities, we hypothesized that the blended essential oil should have a positive impact on human selective attention.

Negative priming (NP) is one of the most influential paradigms for measuring selective attention in cognitive psychology [24–26]. In the typical NP paradigm, a target and a distractor are displayed in the prime trial, followed by a similar probe trial. Participants are instructed to respond to the target and ignore the distractor. In such a task, NP refers to the difference in increased errors and response time (RT) between the probe target and the prime target. One frequently observed event-related potential (ERP) concerning visual identity NP is P300, which has been widely studied as a marker of attentional allocation and the updating of resources needed for stimulus evaluation in a variety of cognitive tasks [27, 28]. Some researchers have reported greater P300 amplitude in the NP condition than in the control condition in selective attention tasks [29, 30], implying that more attentional resources are engaged in NP trials. By contrast, lower amplitudes of P1 and N1 have been observed in the NP condition than in the control condition [31]. As the early sensory aspects of stimulus processing, the reduced amplitudes of P1 and N1 may indicate that sensory refractoriness is more pronounced for the control than for the NP condition. Previous findings have indicated that some brain regions are not only more activated but also more functionally connected when attentional processes are recruited [32].

In the present study, we investigated changes in selective attention induced by the blended essential oil and in related brain activity, using the ERP technique. We hypothesized that, at the behavioral level, the blended essential oil would decrease participants' RT in the NP condition. Correspondingly, at the neural level, P300 amplitude would be smaller in the frontal, parietal, and occipital areas in the same condition. Moreover, it was believed that interaction between distant brain areas enabled the flexible performance of cognitive tasks [33]. We speculated that intrabrain functional connectivity during the NP condition of the blended essential oil group is higher than that of the control group. Thus, the objective of this study was to use ERP to find out how the blended essential oil affected humans' performance on the selective attention task.

## 2. Materials and Methods

### 2.1. Participants

Forty-two undergraduates with a mean age of 21.3 ± 2.7 years (*M* ± SD) were recruited for the study. The participants were randomly assigned to the oil-treatment group or the control-treatment group by using a computer-generated list of pseudorandom integers between 1 and 2 (inclusive). Due to a misunderstanding of the task's requirements, head movements, and program malfunctions, there were 28 valid participants in total, 14 per group. All participants reported that they were in good health and did not have any preference or smell aversion to the blended essential oil, as determined by a pretest. Exclusion criteria included any history of brain diseases, psychiatric disorders, allergies to any essential oil or drug, cardiovascular disease, respiratory disease, or decrements in sense of smell. In addition, participants were asked to refrain from alcohol for a minimum of 12 hours and to have a good sleep of at least 6 hours prior to the experiment. Before the experiment, written informed consent was obtained from each participant. After completing the task, they received the corresponding remuneration. The study procedure was approved by the University Committee on Human Research Protection, East China Normal University.

### 2.2. Essential Oil Preparation

The blended essential oil, obtained from Daikin (China) Investment Co., Ltd. (Shanghai, China), was mainly composed of 1,8-cineole, 3-carene, *β*-pinene, *β*-caryophyllene, and carnosic acid. To maintain uniform concentrations of the blended essential oil throughout the room, several measures had to be taken before the experiment. The interval between the diffusion of the essential oil and the beginning of the task was about 30 minutes, which was enough for the blended essential oil to spread evenly around the room. The size of the EEG measurement room was 16 m^2^, with identical humidity (50%–65%) and a constant temperature (22°C). Two drops of the blended essential oil (about 0.1 ml) were added to fragrance tester strips and then diffused into the air by the special forced air systems provided by the Daikin (China) Investment Co., Ltd. These settings ensured that participants completed the task in an identical environment and that the blended essential oil was used to the best effect.

### 2.3. Stimuli

The stimuli were composed of letters and numbers. For the letter, the uppercase version of the consonants M, N, P, Q, R, T, and X was randomly selected. For the number, the digits 1, 2, 3, 4, 6, 7, and 8 were used. In each trial, the target stimulus appeared in the center of the screen and the distractor was randomly presented in one of four locations (over, under, left, or right) away from the center of the screen. The presentation of the stimuli and recording of the responses were controlled by E-prime software (Psychology Software Tools Inc. 2). All stimuli were presented on a high-resolution computer screen that was positioned at a distance of approximately 70 cm from the viewer.

### 2.4. Procedures

A practice session took place before the formal experiment. If the subjects had no questions about the experiment after the practice, they were able to start the experiment. Each trial began with a 500 ms fixation, followed by a presentation of the prime stimuli. Participants had to judge whether the target stimuli presented in the center of the screen were a letter or a number. If the response target was a letter, participants were required to press the “F” key; if it was a number, they were to press the “J” key. After this, the same response was to be made for the probe stimuli.

**Figure 1:**
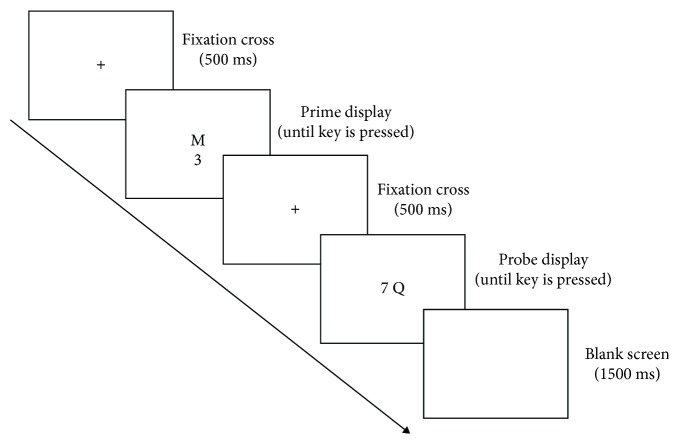
The experimental procedure.

Each trial consisted of prime stimuli and probe stimuli, resulting in four different types of combination: letter-letter, letter-number, number-number, and number-letter. The letter-number combination and number-letter combination were grouped as the NP condition. The letter-letter combination and number-number combination fell into the same category, namely, the positive priming condition, as the control condition. The task included 80 trials in total, 20 trials for each type, presented in a pseudorandom way (see Figure 1).

### 2.5. EEG Data Acquisition

EEGs were recorded using the Geodesic Photogrammetry System™ (GPS) with 64 channels (Electrical Geodesic Inc., USA). The electrode cap was in accordance with the international 10-10 system. We also recorded vertical and horizontal electrooculograms (EOGs) to control for eye movements and eye blinks. Electrode impedance was kept under 50 k*Ω* for all recordings. EEGs were continuously recorded at a sampling frequency of 250 Hz.

### 2.6. EEG Data Analysis

EEG data analysis was conducted using EEGLAB [34] and custom MATLAB (MathWorks Inc., Natick, MA) scripts. In the EEG data preprocessing, we applied a 0.1 Hz high-pass filter, a 30 Hz low-pass filter, and a 50 Hz notch filter consecutively to remove noise. EEG data were rereferenced using a common average reference. Then, to remove ocular artifacts (eye blinks and eye movements) and muscular artifacts, independent component analysis (ICA) was applied to the EEG data [34]; the ADJUST plug-in was then used to remove the ICA components that contained the artifacts [35]. EEG during each trial was segmented from 200 ms before the onset of the probe stimulus display to 500 ms. Epochs were the baseline corrected to the 200 ms prestimulus baseline.

P300 amplitude was identified as the largest positive peak between 300 and 500 ms after the onset of the stimulus. We selected two electrodes of C3 and C4 in the frontal-central area, three electrodes of Pz, P3, and P4 in parietal area, and three electrodes of Oz, O1, and O2 in the occipital area for P300. A repeated measures ANOVA was performed on the mean P300 peak amplitude for probe stimuli with the priming condition as the within-subject variable (negative prime or control) and treatment (essential oils or no essential oils) as the between-subject variable. The false discovery rate (FDR) correction was conducted on the *p* values of the interaction effect to avoid a “Type I” error.

### 2.7. Intrabrain Functional Connectivity Analysis

Synchronization is usually measured between two signals across time and used to measure intra-/interbrain functional connectivity. The EEG dynamics was then quantified with the time-varying phase-locking value (PLV) which provided an index of neural synchrony. After a band pass filtering of the EEG signal, we applied a Hilbert transform to calculate the instantaneous phase *ϕ* of each electrode signal. The PLV formula for two channels (such as *p* and *q*) is given by

(1) $$\begin{array}{c} {\text{PLV}}_{p,q}=\frac{1}{T}\left|\overset{T}{\underset{t=1}{\sum }}\exp^{i\left(\phi p^{\left(t\right)}-\phi q^{\left(t\right)}\right)}\right| \end{array}$$

This formula was calculated across all epochs of one subject, with *T* representing the number of trials. Four frequency bands were chosen for analysis: theta band (4–7 Hz), alpha band (8–12 Hz), beta band (13–30 Hz), and gamma band (31–48 Hz). The intrabrain functional connectivity was characterized by an increase of the average PLV values in the anatomical coupling on the oscillatory activity among electrodes. All PLV values among electrode pairs were subjected to a *t*-test and were corrected using the false discovery rate (*p* < 0.05, FDR correction) [36].

## 3. Results

### 3.1. Behavioral Results

**Figure 2:**
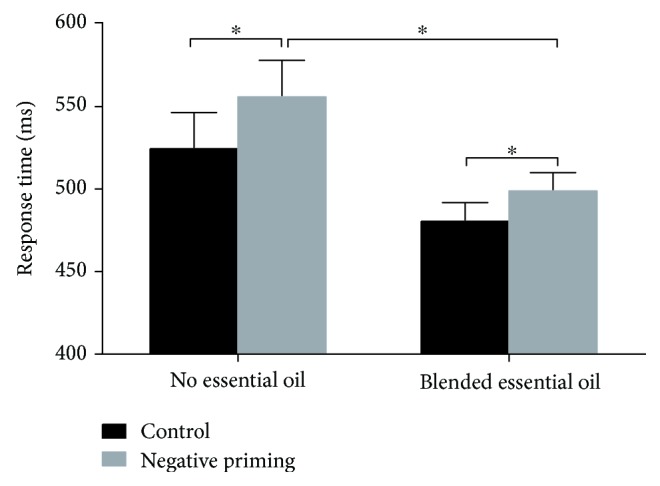
Behavioral results. The mean response times (RTs) of two priming conditions (control and negative priming) for different groups (no essential oil and blended essential oil). ^∗^*p* < 0.05.

Trials containing incorrect responses or those with response times (RTs) deviated more than two standard deviations from an individual's mean RT were excluded for further analysis. The results of a two-way ANOVA on response time showed a main effect of priming condition (see Figure 2, *F* (1, 26) = 17.89, *p* < 0.001, *η*^2^ = 0.41). The response time in the negative priming condition was significantly longer compared with the control condition. These results indicated a reliable negative priming effect. A significant difference was also found for the main effect of the treatment (see Figure 2, *F* (1, 26) = 4.80, *p* = 0.038, *η*^2^ = 0.16). The blended essential oil group showed a shorter response time compared to the no essential oil group.

### 3.2. EEG Results

#### 3.2.1. P300 in the Frontal-Central Area

**Figure 3:**
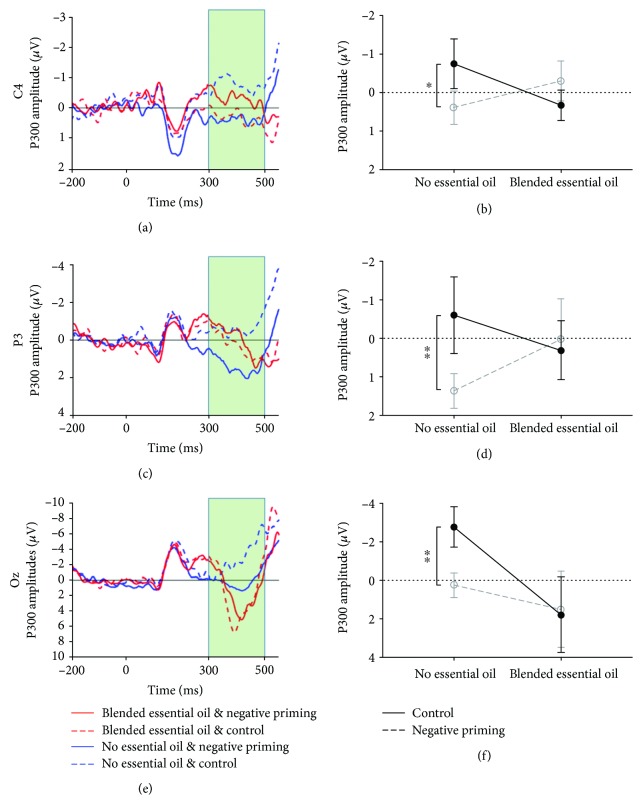
P300 waveforms and mean P300 amplitudes. (a, b) The amplitude in the time window 300–500 ms after the probe stimulus onset for the P300 component at C4. (c, d) The amplitude in the time window 300–500 ms after the probe stimulus onset for the P300 component at P3. (e, f) The amplitude in the time window 300–500 ms after the probe stimulus onset for the P300 component at Oz. ^∗^*p* < 0.05 and ^∗∗^*p* < 0.01.

A repeated-measures ANOVA, used to examine the mean P300 in the frontal-central area, revealed a significant interaction effect of the priming condition and the treatment at C4 (see Figure 3(a), *F* (1, 26) = 6.34, *p* = 0.04, FDR correction, *η*^2^ = 0.20). The simple effects analysis showed that the mean P300 amplitude of the negative priming trials was greater than that of control trials for the group without the blended essential oil (see Figure 3(b), *t* (13) = 2.18, *p* = 0.048, FDR correction, Cohen′s *d* = 0.68), whereas there was no such effect in the oil-treatment group. The interaction of treatment by priming condition did not reach a significant level at C3 (*p* > 0.05, FDR correction).

#### 3.2.2. P300 in the Parietal Area

Similarly, a repeated-measures ANOVA analysis of the mean P300 also revealed a significant interaction effect at P3 in the parietal area (see Figure 3(c), *F* (1, 26) = 6.82, *p* = 0.04, FDR correction, *η*^2^ = 0.21). The simple effects analysis showed that the negative priming condition induced greater P300 compared to the control condition in the no essential oil group (see Figure 3(d), *t* (13) = 2.96, *p* = 0.01, FDR correction, Cohen′s *d* = 0.69). However, no such difference was observed in the blended essential oil group. No interaction effect was observed at Pz or P4 (*p* > 0.05, FDR correction).

#### 3.2.3. P300 in the Occipital Area

The same ANOVA analysis showed a significant interaction effect of priming type and treatment on P300 at Oz (see Figure 3(e), *F* (1, 26) = 6.77, *p* = 0.04, FDR correction, *η*^2^ = 0.21). In detail, the mean P300 amplitude of the negative priming trials was greater than control trials in the group without the blended essential oil (see Figure 3(f), *t* (13) = 2.79, *p* < 0.048, FDR correction, Cohen′s *d* = 0.93), but not in the blended essential oil group. Nevertheless, no significant interaction effect was found at O1 or O2 (*p* > 0.05, FDR correction).

### 3.3. Intrabrain Functional Connectivity

**Figure 4:**
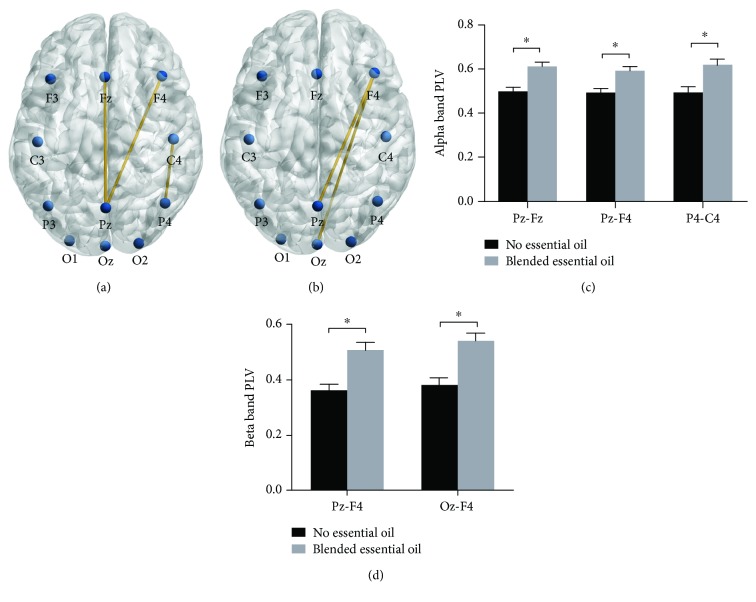
Intrabrain functional connectivity. (a) The increased intrabrain PLVs in alpha band in the blended essential oil group compared to the no essential oil group. (b) The increased intrabrain PLVs in beta band in the blended essential oil group compared to the no essential oil group. (c) The comparison of mean alpha band intrabrain PLVs in Pz-Fz, Pz-F4, and P4-C4. (d) The comparison of mean beta band intrabrain PLVs in Pz-F4 and Oz-F4. ^∗^*p* < 0.05, FDR correction.

The EEG data was then analyzed with the Phase Locking Value (PLV) which provided a measurement to quantify functional connectivity between two areas in the brain. The PLV analysis showed that essential oils induced stronger intrabrain functional connections during performance of the selective attention task. Specifically, higher PLVs in alpha band were found in Pz-Fz (*t* (13) = −4.05, *p* = 0.01, Cohen′s *d* = 1.53), Pz-F4 (*t* (13) = −4.20, *p* = 0.02, Cohen′s *d* = 1.59), and C4-P4 (*t* (13) = −3.56, *p* = 0.03, Cohen′s *d* = 1.35) in the blended essential oil group compared to the no essential oil group (see Figures 4(a) and 4(c)).

Meanwhile, increased PLVs in the beta band were observed within the Pz-F4 (*t* (13) = −4.23, *p* = 0.01, Cohen′s *d* = 1.60) and Oz-F4 (*t* (13) = −4.20, *p* = 0.01, Cohen′s *d* = 1.59) (see Figures 4(b) and 4(d)) in the blended essential oil group compared to that in the no essential oil group. Thus, the blended essential oil could strengthen functional connectivity between the frontal lobe and parietal lobe in both alpha band (Pz-Fz, Pz-F4, and P4-C4) and beta band (Pz-F4 and Oz-F4).

## 4. Discussion

In the present study, we used the ERP technique to examine the effect of blended essential oil on human selective attention and the neural basis of this effect. Behavioral results showed that the blended essential oil was able to improve participants' performance in the NP task by shortening RT in the NP trials. ERP results revealed the interaction effect of the treatment and the priming condition. The P300 amplitudes at C4, P3, and Oz were significantly higher in the NP condition than in the control condition for the “no essential oil” group. However, this effect disappeared when participants were exposed to the blended essential oil. A PLV analysis showed stronger intrabrain synchrony in the alpha band (Pz-Fz, Pz-F4, and P4-C4) and beta band (Pz-F4 and Oz-F4) in the oil-treatment group than in the “no essential oil” group. Our findings provided both behavioral and neural evidence for the positive effect of blended essential oil on human selective attention.

First, the behavioral results showed that the mean RT of the NP condition was longer than that of the control condition in both groups, demonstrating a NP effect consistent with previous works [37–39]. The quicker response for the NP trials in the blended essential oil group reflected better performance in selective attention. Together, these findings support our hypothesis that inhalation of the blended essential oil can improve selective attention.

Second, as reported in previous studies [37, 40], the NP effect was accompanied by an increase in P300 amplitude in the “no essential oil” group. However, the same result was not found in the oil-treatment group. P300 amplitude is often interpreted as an index of updating resources and mental efforts that are needed for stimulus in a variety of cognitive tasks [41]. Thus, it is generally assumed that the larger P300 amplitude corresponds to a greater allocation of attention. Our ERP findings therefore suggest the neural mechanism through which the blended essential oil influenced human selective attention. That is, the blended essential oil was able to facilitate processing and decrease the mental effort in the NP by affecting P300 amplitude.

Third, the inhalation of blended essential oil can significantly increase the PLV in the alpha and beta bands, which resembled the behavioral pattern of accuracy very well. The PLV provided a frequency-specific synchronization measure between two signals across time [42] and interaction between neural assemblies [43]. It is believed that this interaction between distant brain areas may promote the formation exchange of transient functional networks, enabling the flexible performance of cognitive tasks [33, 43, 44]. This finding was also consonant with previous ERP studies indicating that increased phase locking between the parietal cortex and occipital cortex during attention is associated with enhanced performance [45]. Thus, the greater intrabrain alpha and beta band synchronization in the blended essential oil group may be relevant for the successful engagement of selective attention.

The results of this study show that exposure to the aroma of a blended essential oil can significantly enhance performance on NP tasks in humans by modulating brain wave activity and brain functional connections. Our findings partly support prior observations that some essential oils significantly improve the psychophysiological conditions of humans, such as attention, working capacity, mood, and stress. The main chemical components of the blended essential oil used in this experiment are 1,8-cineole, 3-carene, *β*-pinene, and *β*-caryophyllene, which have been proven to have a stimulating effect on sympathetic activity. It may be beneficial for the brain to remain active and vigilant [46], which may enhance the cognitive performance of humans. In addition, an increase of reactive oxygen species (ROS) in the brain tissues of humans can lead to neurotoxicity damage and oxidative stress [47]. Carnosic acid and 1,8-cineole can attenuate oxidative injury by inhibiting ROS production and increasing endogenous antioxidant compounds [48, 49]. The present findings indicate that the blended essential oil's effects on task performance are mainly associated with the stimulation of sympathetic activity and subsequent alteration of brain activity and function.

Our study is not without limitations. First, it was difficult for us to identify the precise brain regions for selective attention because of the low spatial resolution of the EEG technique. Future research could investigate the neural mechanisms underlying selective attention by combining fMRI and EEG techniques. Second, PLV has been challenged for its spurious hyperconnections [50]. Measures and analyses that are more robust should be applied in future studies to consolidate the current findings.

In summary, the blended essential oil appears to improve performance in selective attention tasks by shortening response times in NP trials, modulating P300 amplitude, and increasing intrabrain phase synchronization in the frontal-parietal networks. Our findings suggest that the blended essential oil could be used as a stimulant to improve human selective attention.

## 5. Conclusions

In summary, the blended essential oil appears to improve performance in selective attention tasks by shortening response times in NP trials, modulating P300 amplitude, and increasing intrabrain phase synchronization in the frontal-parietal networks. Our findings suggest that the blended essential oil could be used as a stimulant to improve human selective attention.

## Data Availability

The data used to support the findings of this study are available from the corresponding author upon request.
